# The Results of the Antitoxin Treatment of Diphtheria in Germany

**Published:** 1899-07-01

**Authors:** 


### THE RESULTS OF THE ANTITOXIN TREATMENT OF DIPHTHERIA IN GERMANY.

Protests have been raised in many quarters against the statistics collected by the officials of the Metropolitan Asylums Board in regard to the results of treatment by antitoxin, but although there may be a certain amount of force in some of these objections, a careful re-examination of the statistics has convinced us that, whatever allowance one may have to make for possible errors, there is a substantial balance in favour of antitoxin. Such being the case it is satisfactory to find that this conclusion is supported by certain returns recently issued by the Imperial Board of Health of Prussia. They have published two maps of that country, coloured to show the mortality from diphtheria in the various parts of the kingdom in the periods 1885-94 and 1895-97 respectively. These periods represent the time before and the time after the general introduction of antitoxin in the treatment of diphtheria, it being only since the year 1894 that the supply has been unlimited and its use practically imperative. Now during the first-mentioned period the mortality averaged 15·5 for the whole country, while it fell at once in 1895 to 9·0 per cent., in 1896 to 7·6 per cent., and in 1897 to 6·2 per cent. This shows that a very sudden and a very large reduction in the mortality from the disease was at least coincident with the introduction and wide acceptance of the new method of treatment. But what is of great importance, as tending to show that it was the treatment which was the effective cause of this improvement, is the tale told by the maps, which show that the proportionate incidence of the disease on the different parts of the country was not materially altered. Within its bounds are the remains of several nationalities or races variously intermingled, and it is found that according to the preponderance of the Teutonic or the Slavic elements in the population, or, in other words, according to the preponderance of industry, thrift, and cleanliness, or of poverty, indolence, and dirt, so is the rarity or the prevalence of diphtheria in the different areas in question. When, then, we see that notwithstanding the great diminution in the prevalence of diphtheria which has taken place all over this great area, its relative incidence on the difference parts has remained practically unchanged, we must either imagine the intrusion of some occult epidemic influence of so strange a nature as to have affected at the same time such widely different races, notwithstanding their very different social and sanitary conditions, or we must attribute the general lowering of the mortality from the disease to what we know did happen at that very time, and did affect every portion of the country under Prussian rule, whether inhabited by Slavs or Germans, namely, the introduction of the antitoxin treatment, which was then placed at the disposal of the profession throughout Prussian territory. This, again, may not be proof of the efficacy of antitoxin, but it is very strong confirmatory evidence.

